# 

**Published:** 1899-06-03

**Authors:** 


					THE HOSPITAL. "The Standard of highest purity."?Lancet, .TUNE 3rd, 1899.
(V CADBURY'S V fei CADBURY'S
cocoa WL m m cocoa
ABSOLUTELY PURE. 6? " ~~-*y NO DRUGS OR ALKALIES USED.
Ur-iSj?^Si.flscotv Western Infirmary
No. 66;. Vol. XXVI. OfaJSgJiO Saturday,
June 3rd, 1899.
wIoTh SsPfrTti
Terms,"]
see p. 160. J
["Subscription Terms,"] pRICE TWOPENCE.

				

